# Addressing Longevity Risk: How Best to Annuitize Defined Contribution Assets?

**DOI:** 10.1093/geroni/igaa057.2395

**Published:** 2020-12-16

**Authors:** Alicia Munnell, Gal Wettstein, Wenliang Hou

**Affiliations:** 1 Boston College, Chestnut Hill, Massachusetts, United States; 2 Center for Retirement Research at Boston College, Chestnut Hill, Massachusetts, United States

## Abstract

Unlike defined benefit pensions, 401(k) plans provide little guidance on how to turn accumulated assets into income. The key risk that retirees face is outliving their assets. Insurance against such risk is available through several routes, including immediate annuities, deferred annuities, and additional Social Security through delayed claiming. Under this Social Security bridge option, participants would tap their 401(k) for payments equal to their Social Security to delay claiming. This paper compares these three options in simulations against a baseline in which no assets are used to obtain lifetime income. In each option, assets not allocated to purchasing lifetime income are consumed following the Required Minimum Distribution rules. The analysis finds that, when market and health shocks are included alongside longevity uncertainty, the Social Security bridge option is generally the best for households with median wealth. Wealthier households can benefit from combining the bridge option with a deferred annuity. Part of a symposium sponsored by the Economics of Aging Interest Group.

